# Bacteriophage-Based Methods for Detection of Viable *Mycobacterium avium* subsp. *paratuberculosis* and Their Potential for Diagnosis of Johne's Disease

**DOI:** 10.3389/fvets.2021.632498

**Published:** 2021-03-11

**Authors:** Irene R. Grant

**Affiliations:** School of Biological Sciences, Institute for Global Food Security, Queen's University Belfast, Belfast, United Kingdom

**Keywords:** Johne's disease diagnosis, *Mycobacterium avium* subsp. *paratuberculosis*, phage-based detection methods, phage amplification assay, phagomagnetic separation, viability test

## Abstract

Bacteriophage-based methods for detecting *Mycobacterium avium* subsp. paratuberculosis (MAP) are a potential new approach for diagnosis of Johne's disease (JD). The basis of these tests is a mycobacteriophage (D29) with a lytic lifecycle that is able to infect a range of *Mycobacterium* spp., not just MAP. When added to a test sample, the phages will bind to and infect mycobacterial cells present. If the host mycobacterial cells are viable, the phages will take over the metabolic machinery of the cells to replicate and produce multiple copies of themselves (phage amplification), before weakening the host cell walls by enzyme action and causing cell lysis. Cell lysis releases the host cell contents, which will include ATP, various enzymes, mycobacterial host DNA and progeny D29 phages; all of which can become the target of subsequent endpoint detection methods. For MAP detection the released host DNA and progeny phages have principally been targeted. As only viable mycobacterial cells will support phage amplification, if progeny phages or host DNA are detected in the test sample (by plaque assay/phage ELISA or qPCR, respectively) then viable mycobacteria were present. This mini-review will seek to: clearly explain the basis of the phage-based tests in order to aid understanding; catalog modifications made to the original plaque assay-based phage amplification assay (FASTPlaqueTB™) over the years; and summarize the available evidence pertaining to the performance of the various phage assays for testing veterinary specimens (bovine milk, blood and feces), relative to current JD diagnostic methods (culture, fecal PCR, and blood-ELISA).

## Introduction

Paratuberculosis, or Johne's disease (JD), caused by *Mycobacterium avium* subsp. *paratuberculosis* (MAP), is a chronic enteritis of domesticated ruminant animals that is very much a hidden and often endemic problem for farmers worldwide (1, 2). It is widely acknowledged that the available tests for the diagnosis of JD are imperfect and do not detect all MAP infected animals (3). Consequently, JD control efforts based on fecal culture and serum- or milk-ELISA results have not been as effective as national governments would have liked (2). Culture remains the definitive diagnostic test for JD, but takes too long to deliver results; it has been the only method available to confirm the presence of viable MAP, i.e., the infectious agent, in veterinary specimens for many years. Phage-based methods are a relatively recent potential addition to the JD diagnostic toolbox; their development being principally progressed by two research groups in the United Kingdom (Professor Catherine Rees' group at University of Nottingham and the author's group at Queen's University Belfast) since the mid 2000s. Currently, other than culture and some viability dye-based qPCR methods (4–6), phage-based tests represent the only other means of specifically detecting and distinguishing viable MAP. This mini-review will seek to, firstly, clearly explain the basis of the phage-based tests in order to aid understanding of how such tests work. Secondly, it will catalog modifications made to the original plaque assay-based phage amplification assay (FAST*Plaque*TB™) over time in an effort to simplify the assays and make them more user-friendly. Finally, the available evidence pertaining to the performance of the phage assays for testing veterinary specimens (bovine milk, blood, and feces), relative to current JD diagnostic methods (culture, fecal PCR, and blood-ELISA), will be summarized.

## How Do Phage-Based Tests for Detection of Viable MAP Work?

**Figure 1:**
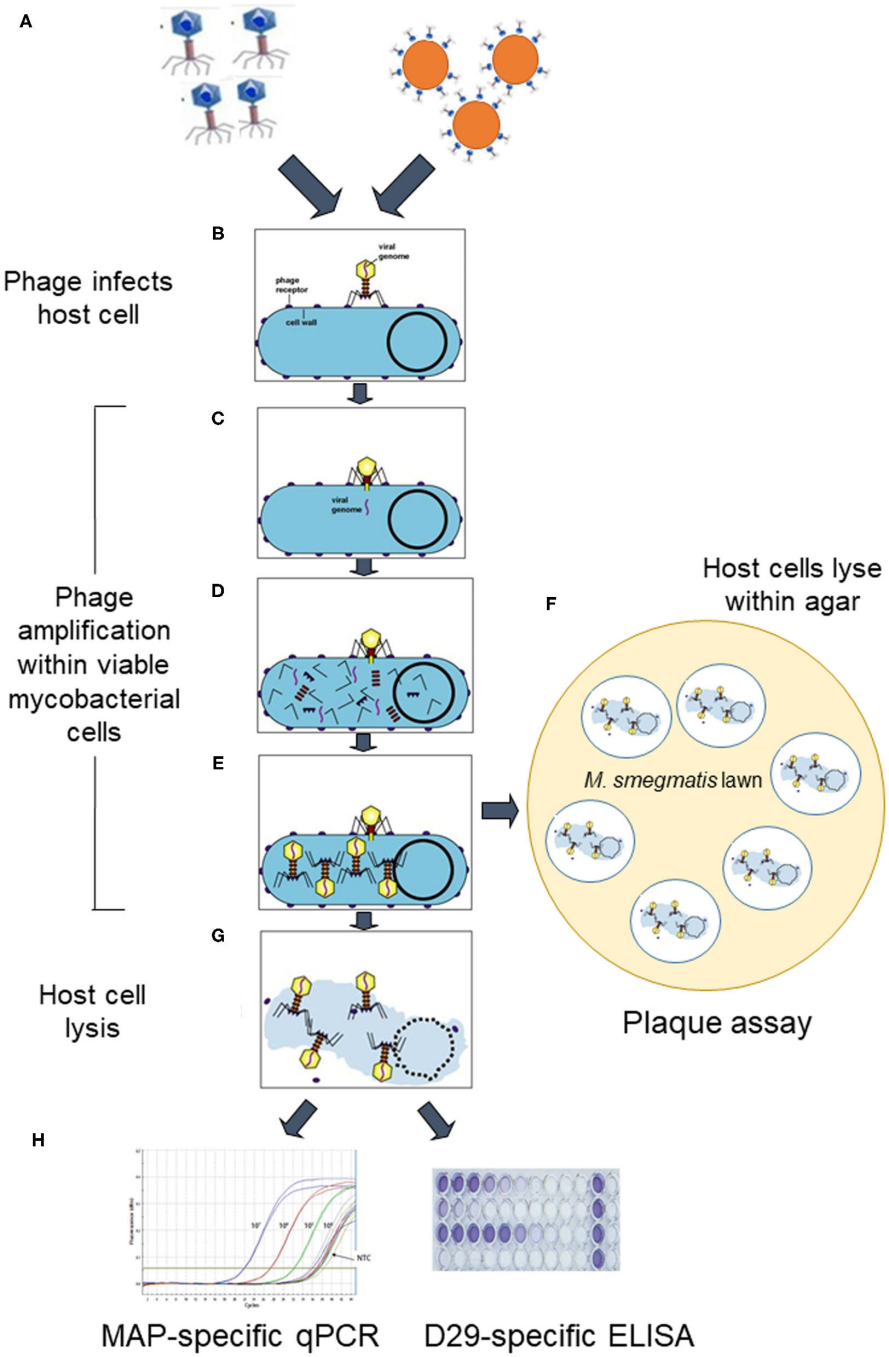
Schematic overview of how lytic phage-based assays for detection of viable *Mycobacterium avium* subsp. *paratuberculosis* work. D29 phages are added to the sample as either free phages or as phage-coated paramagnetic beads (step **A**). They specifically bind irreversibly to host mycobacterial cells (step **B**) in the sample and then infect the cells by injecting their genome (step **C**). The host cell's machinery is hi-jacked to reproduce phage component parts (step **D**), which assemble into mature phages within the host cell (step **E**). At this point the phage-treated sample is either plated in agar before mycobacterial cells burst to release progeny phages which infect a *Mycobacterium smegmatis* lawn and form zones of clearing (plaques) (step **F**), or incubation proceeds until after burst of mycobacterial cells occurs due to action of phage endolysins in suspension (step **G**). When cells burst, host cell DNA and progeny phages are released, which can be detected by a MAP-specific qPCR or D29-specific ELISA, respectively (step **H**) (Elements of this figure are not to scale and are for illustrative purposes only.). Certain elements of this figure are used under licence BY-NC-SA 3.0 from Kaiser (11) 10.7A: The Lytic Lifecycle of Bacteriophages. Biology LibreTexts™.

A mycobacteriophage with a lytic (virulent) lifecycle, known as D29 (7), has been employed for all MAP phage assays developed to date. D29 has a broad host range amongst the *Mycobacterium* spp., including *M. tuberculosis, M. bovis, M. avium, M. scrofulaceum*, and *M. ulcerans* (8–10). Hence, a test based on D29 phages alone will never be specific for MAP; although it will be specific for viable mycobacterial cells (i.e., host cells with functioning metabolism that facilitate replication of the infecting phage within them). To add specificity for MAP, PCR or qPCR have needed to be applied as a confirmatory final step in the vast majority of published phage-based methods. The different published phage-based tests all have at their core phage amplification (multiplication) within viable host mycobacterial cells, as illustrated in Figure 1. Differences between phage-based tests for detection of MAP principally relate to: (1) how the mycobacterial cells are prepared prior to addition of phages; (2) how the phages are added to the test sample; and (3) what is detected once mycobacterial cells present in a sample lyse (burst) due to phage action, i.e., progeny phages and/or MAP DNA. Each of the published phage-based methods developed for detection of viable MAP will be briefly described, categorized by what they detect.

## Methods to Detect Progeny Phages After Phage Amplification and MAP Cell Lysis

### Plaque Assays

The starting point for phage-based methods for detection of viable MAP was when the commercially available FASTPlaqueTB™ assay (Biotec Laboratories Ltd., Ipswich, UK), originally developed for detection of *Mycobacterium tuberculosis* complex in sputum for human Tuberculosis diagnosis (12), was successfully applied with minimal adaptation to detect viable MAP in milk by Stanley et al. (13). This test is a plaque assay which involves fast-growing *Mycobacterium smegmatis* cells to provide the bacterial lawn in which zones of clearing (plaques) form, as a consequence of phage-infected MAP cells bursting within the agar and releasing D29 phages which then repeatedly infect and lyse nearby *M. smegmatis* cells to form plaques during an overnight incubation (Figure 1). However, because the D29 mycobacteriophage involved is not specific for MAP, Stanley et al. (13) added in plaque harvesting, DNA extraction (by heating agar plaques) and IS900 PCR (termed plaque PCR) steps to achieve specificity for MAP. Stanley et al. (13) termed their test the Phage-PCR assay. Subsequently, the use of Zymoclean DNA Clean and Concentrator columns (Zymo Research, Irvine, CA, USA, or similar) to extract DNA from plaques, rather than simple boiling of plaques, was recommended by the Nottingham research group in order to increase the detection sensitivity of the Phage-PCR assay (14, 15). This Phage-PCR assay is marketed as the Actiphage® Core 2-day assay (PBD Biotech Limited, Thurston, Suffolk, UK). It has been used by the Rees research group for many years to test for viable MAP in raw (16) and pasteurized milk (17), other dairy products including infant formula and cheeses (18, 19), and in cattle blood (14). The Phage-PCR assay has also been used for detection of *Mycobacterium bovis* in cattle blood (15, 20).

Altic et al. (21) and Donaghy et al. (22) applied the original FASTPlaqueTB™ assay to detect and enumerate viable MAP in milk after UV treatment, and both groups observed that plaque numbers did not correlate well with MAP colony counts. Foddai et al. (23) optimized the conditions of the original phage amplification assay to achieve accurate enumeration of viable MAP in milk. This was considered to be important because even though the test is more rapid than culture unless the phage-based test is able to accurately indicate the number of viable MAP the true story about prevalence of viable MAP in bovine milk or blood will not be uncovered. The main changes made to the test protocol were an extended incubation time from 1 to 3.5 h before plating with *M. smegmatis* mc^2^ 155 and molten agar, and virucide treatment 2 h into this incubation period rather than just before plating (23).

Subsequently, Foddai et al. (24) inserted a peptide-mediated magnetic separation (PMS) step (25) to selectively separate MAP cells from complex sample matrices and concentrate them into a smaller volume in front of the optimized phage amplification assay (23). The PMS step gives the assay greater specificity for MAP than the earlier Phage-PCR or optimized phage assays. The Peptide-mediated magnetic separation (PMMS)-phage assay referred to by Swift et al. (14) is the same PMS step linked to the Phage-PCR method rather than to the optimized phage amplification assay. Subsequent tweaks to milk sample preparation protocols and application of the PMS-phage assay to test naturally contaminated milk samples were reported (26, 27), but no further changes were made to conditions of the optimized PMS-phage assay *per se*.

### D29-Specific ELISA

In an effort to achieve a more rapid phage-based test for viable MAP, Stewart et al. (28) spent time producing a D29-specific polyclonal antibody to form the basis of a competitive ELISA assay to be used after phage amplication, rather than applying the plaque assay. The new immunoassay, which still included PMS to separate and concentrate MAP cells from a milk or feces sample first, was called the PMS-Phage-ELISA assay (28). Although the PMS step added considerable specificity for MAP detection to the overall assay, some non-MAP mycobacterial cells (e.g., *M. bovis* or environmental mycobacteria) could potentially be captured from naturally contaminated samples, and hence all of the D29 phages detected by the ELISA may not necessarily be due to viable MAP cells. A working assay was achieved that was quicker than the PMS-phage assay (24 h instead of 48 h) and that had good detection sensitivity, however no further work on the PMS-phage-ELISA assay was ever reported.

## Methods to Detect MAP DNA After MAP Cells Have Lysed Due to Phage Action

Two rapid, 1-day phage- and qPCR-based tests for viable MAP have been reported most recently - the Actiphage® Rapid assay (15) and the Phagomagnetic separation (PhMS)-qPCR assay (29). The Actiphage® technology is subject of a patent [(30), PCT/GB2014/052970], and the Actiphage® Rapid assay is commercially available (PBD Biotech Limited). The PhMS-qPCR assay is patent pending [(31), PCT/EP2020/076632], but not yet a commercial test. Whilst the two tests may seem similar, the latter has a different *modus operandi*. Phages are added to the test sample bound to paramagnetic beads rather than as a free phage suspension, as in the Actiphage Rapid® assay. The D29 phages are attached to tosylactivated paramagnetic beads by covalent bonding with capsid (head) proteins, so that tails are orientated outwards to permit binding between phage and MAP cell surface. The phage-coated paramagnetic beads facilitate physical separation of phage-captured MAP cells from potentially inhibitory sample constituents by means of a magnet. Once the bead-bound D29 phages attach to MAP cells, they inject their DNA to infect the host MAP cells and initiate phage amplification (the lytic cycle) within viable cells only. The magnetic beads remain attached to the MAP cells facilitating subsequent washing of the bead-cell complexes, and then resuspension of the beads in a small volume (50 μl) of broth. When the D29 phage-infected mycobacterial cells subsequently burst from the inside out due to phage enzyme action, DNA is released into this small volume and no further DNA extraction or purification is necessary prior to its use as template DNA for MAP-specific Taqman qPCR (6). In contrast, purification of mycobacterial DNA using extraction columns is recommended for the Actiphage Rapid® assay to maximize detection sensitivity (15). Supplementary Table 1 compares the steps involved for the two rapid phage- and qPCR-based assays when testing 50 ml milk. The MAP detection capabilities of these two rapid phage-based tests for viable MAP have yet to be directly compared.

## What Does a Phage Assay Positive Result Mean?

There appears to be a degree of misunderstanding amongst MAP researchers about what a phage assay positive result means, and also considerable skepticism about positive phage assay results that are not supported by parallel culture positive results, or other positive JD diagnostic test result. In theory, the presence of a plaque is due to a single viable mycobacterial cell or a clump of viable mycobacterial cells bursting within the agar, and the progeny phages released infecting and repeatedly bursting *M. smegmatis* cells in the surrounding bacterial lawn. In practice, potential false positive results may arise with plaque-based phage assays due to ineffective viricide treatment, meaning that some plaques would be due to non-inactivated D29 phages, or some cells bursting before plating in agar happens, also releasing D29 phages that will interact with *M. smegmatis* lawn to form plaques. Early adopters of the phage-PCR assay or PMS-phage assay have run into such issues, and have found the multiple steps and transfers involved, the timed incubation steps, the need for molten agar and an *M. smegmatis* culture, and one or two overnight incubations tedious (32). In my experience of transferring our optimized Phage assay or PMS-phage assays to a number of other laboratories, this has rarely proved to be a straightforward process and a lot of follow-up troubleshooting activity has ensued. It seems that many years of practice with the plaque assays at University of Nottingham and Queen's University Belfast make perfect, and that some degree of proficiency in their use must be acquired before reliable results can be consistently obtained in other laboratories.

The two most recently published 1-day phage assays (Actiphage® Rapid and PhMS-qPCR assays) are no longer reliant on plaque assays and subsequent plaque PCR for confirmation of a positive result. These tests have clear advantages compared to plaque assay-based tests, not just in terms of speed of results but also in terms of greater sensitivity of detection and more accurate viable MAP counts being obtained. This is due to the fact that all viable MAP cells in the sample will contribute DNA for the final qPCR step in the assay, rather than a random selection of 5–10 plaques that may or may not have arisen from lysed MAP cells picked from agar plates. In the author's opinion, these rapid phage-based assays should be less problematic for intending users, given that the test protocols have been streamlined to require fewer manipulations and transfers, are less reliant on accurate incubation times, and have qPCR as the endpoint detection step. Furthermore, many veterinary diagnostic laboratories will already be familiar with qPCR if they carry out fecal qPCR for Johne's or other animal disease diagnosis, for instance.

## Application of Phage-Based Tests for Diagnosis of MAP Infection in Cattle

To date, the University of Nottingham research group has principally focussed on applying their phage-based tests (PMMS-Phage-PCR and Actiphage Rapid® assay) to blood sample from cattle for detection of viable MAP and *M. bovis*. In contrast, the Queen's University Belfast research group has concentrated on applying their methods (PMS-phage assay and PhMS-qPCR) for detection of viable MAP in bulk tank milk and individual cows' milk primarily, but have also tested some bovine feces (24). In my experience, without magnetic separation to remove inhibitory components in feces the phage assay cannot be successfully applied to this specimen type. Table 1 summarizes the findings of published studies relating to detection of viable MAP in naturally contaminated cattle samples. Consistently, more viable MAP positive results are being obtained with the phage-based assays compared to culture, whether performed with prior chemical decontamination or PMS (20, 24, 27). The rapid, 1-day phage- and qPCR-based assays are proving to be more sensitive than either the Phage-PCR or PMS-phage assays, which are plaque assay-based tests (15, 29, 34). Phage assay results are often indicating the presence of viable MAP bacteraemia or MAP shedding in milk and feces in animals that are serum- or milk-ELISA negative (14, 15, 20, 34). O'Brien et al. (33) determined the diagnostic specificity and sensitivity of the PMS-phage assay to be 1.00 and 0.325, respectively. PMS-culture specificity and sensitivity values when applied to the same samples were 0.962 and 0.250, respectively. Only a single herd was used as the “non-infected” cohort during this study and because four animals in that population were fecal culture positive (confirmed by IS900 PCR), the diagnostic specificity estimates may not be accurate.

**Table 1:** Main findings of studies applying phage-based assays to detect viable MAP in naturally infected bovine milk, feces or blood.

**Study**	**Type of phage assay**	**Sample type (no. of samples)**	**Comparator test(s)**	**Main findings**
Foddai et al. (24)	PMS^a^-phage assay	Bulk tank milk (*n* = 44), feces (*n* = 39)	HPC^b^ + culture or PMS-culture (milk), real-time qPCR (feces)	Bulk tank milk: 15/44 (34.1%) samples tested positive by PMS-phage assay, with numbers of viable MAP detected ranging from 1 to 110 PFU^c^/50 ml BTM. 5/44 (11.4%) samples were positive by culture after HPC decontamination or PMS. Feces: 20/39 (51.2%) samples tested positive by PMS-phage assay, with numbers of viable MAP detected ranging from 6 to 41,111 PFU/g. 35/39 (89.7%) feces samples had been positive by RT-qPCR when tested several months previously.
Swift et al. (14)	PMMS^a^-Phage-PCR	Bloods from milk-ELISA positive cattle (*n* = 9, Set A), cattle in JD-free herd (*n* = 5, Set B), and from cattle with strong, intermediate or negative milk-ELISA results (*n* = 10, Set C)	Serum-ELISA, Culture without decontamination	Set A: 9/9 (100%) bloods tested PMMS-phage-PCR positive, with MAP counts ranging from 3 to 35 PFU/ml blood, compared to 8/9 (88.9%) by serum-ELISA. Set B: 0/5 (0%) bloods tested PMMS-phage-PCR positive, same by serum-ELISA. Set C: 8/10 (80%) bloods tested PMMS-phage-PCR positive compared to 4/10 (40%) positive by serum-ELISA and 0/10 (0%) positive by culture.
Botsaris et al. (16)	Phage-PCR	Bulk tank milks (*n* = 225) in Cyprus	HPC + culture	218/225 (96.9%) milk samples yielded plaques, i.e., contained viable mycobacteria. Only 50/225 (22.2%) milk samples tested positive for presence of MAP DNA by plaque PCR. In contrast, just 2/225 (0.9%) milk samples yielded colonies confirmed to be MAP after HPC and culture.
Swift et al. (20)	PMMS-phage assay	Bloods from 4.5 year old cattle that had been orally inoculated with MAP at 3–4 months of age (*n* = 19)	Fecal culture, fecal qPCR and Serum-ELISA	7/19 (37%) blood PBMCs^d^ tested positive by PMMS-Phage-PCR, with low numbers of MAP indicated (2–5 PFU). 2/19 (10.5%) and 1/19 (5.3%) tested positive by fecal culture and serum ELISA, respectively.
Foddai and Grant (27)	PMS-phage assay	Milk from individual cows in a JD affected dairy herd (*n* = 146), and bulk tank milk from Johne's affected dairy farms (*n* = 22).	PMS-IS900 qPCR and PMS-MGIT culture.	Limit of detection (LOD_50%_^e^) of the PMS-phage assay reported as 0.93 MAP cells/50 ml milk. Viable MAP detected in 31/146 (21.2%) milks from individual cows and from 13/22 (59.1%) bulk tank milks by the PMS-phage assay, with numbers of viable MAP detected ranging from 6 to 948 PFU/50 ml. Fewer MAP positive samples detected by PMS-qPCR (Individual: 9.1%, BTM 45.4%) and PMS-culture (Individual: 11.6%, BTM: 50.0%). “Moderate” agreement between PMS-phage assay and PMS-qPCR results for BTM (*p* = 0.0036), “poor to fair” agreement for individual milks (*p* = 0.1695).
O'Brien et al. (33)	PMS-phage assay	Milk from MAP test negative cattle (*n* = 105) and MAP test positive animals (*n* = 40)	Serum-ELISA, Fecal culture, PMS-culture	Diagnostic sensitivity (DSe) and specificity (DSp) of the PMS-phage assay were 0.325 and 1.000, respectively, compared to 0.250 and 0.962 for PMS-culture, and 0.525 and 0.962 for the PMS-phage assay and PMS-culture results combined.
Swift et al. (15)	Actiphage® Rapid assay	Bloods from experimentally MAP infected calves (*n* = 15) and non-infected control calves (*n* = 8)	Phage-PCR, IDEXX ELISA and tissue culture (at necropsy)	MAP infected calves: 13/15 (87%) blood PBMC samples Actiphage® Rapid assay positive and 6/15 (40%) Phage-PCR assay positive. No calves tested MAP positive by either serum-ELISA or tissue culture. Non-infected calves: 2/8 (25%) blood PBMC samples Actiphage® Rapid assay positive. No bloods positive by Phage-PCR or serum-ELISA, and no MAP cultured from tissues. Actiphage® Rapid assay had greater MAP detection sensitivity than original Phage-PCR assay. Limit of detection reported as 1–10 MAP cells/ml blood.
Foddai and Grant (29)	PhMS^f^-qPCR assay	Bulk tank milk (*n* = 100)	None	Limit of detection (LOD_50%_) of the optimized PhMS-qPCR assay reported as 10 MAP cells/50 ml milk (95% CI: 1.20–82.83). 49/100 (49%) bulk tank milks tested PhMS-qPCR positive with number of viable MAP detected ranging from 3 to 126 MAP/50 ml milk.
Foddai et al. (29)	PhMS-qPCR assay	Bulk tank milk (*n* = 392) and individual milks from cows on four MAP-infected farms (*n* = 293)	Milk-ELISA, PMS-culture	Bulk tank milks: Viable MAP detected in 103/392 (26.5%) bulk tank milks by PhMS-qPCR, with MAP levels ranging from 1 to 8,432 MAP/50 ml; <2% of the 392 farms had MAP contamination levels >100 MAP cells/50 ml. Individual milks: 17–24% of animals in four of the above farms showing highest MAP contamination levels in their bulk tank milk tested PhMS-qPCR positive, with MAP levels between 6.7 and 42.1 MAP cells/50 ml. There was no significant correlation between parallel PhMS-qPCR and milk-ELISA results for either BTM or individual milks. When subjected to PMS-culture, 52/61 (85%) PhMS-qPCR positive milks yielded an IS900 qPCR positive Pozzato broth culture.

a *PMS and PMMS, peptide-mediated magnetic separation. Swift et al. (14, 20) used beads coated with peptides as described by Foddai et al. (24)*.

b *Hexadecylpyridinium chloride decontamination*.

c *PFU, Plaque-forming units*.

d *PBMC, peripheral blood mononuclear cells isolated from whole blood before testing*.

e *LOD_50%_ is the microbial analyte concentration (and confidence limits) that corresponds to a 50 % probability of a positive result with the test method*.

f *PhMS, phage-mediated magnetic separation (known as phagomagnetic separation)*.

## Current State-of-Play and What Next

More validation data for the most recent rapid phage- and qPCR-based methods is urgently needed, in order to accumulate a convincing body of evidence demonstrating the tests' performance relative to culture results; although it must be remembered that depending on how culture is carried out it may not be a perfect comparator test. It will be important that follow-up longitudinal studies of animals that have tested phage assay positive (by whichever version of phage-based test applied and whichever sample type tested) but with discrepant serum- or milk-ELISA and fecal qPCR negative results are carried out. Ideally, further work to make the latest rapid phage-based tests higher throughput and more automated, with applicability for testing a broad range of veterinary specimen types, would also be undertaken. The development of more complex phage-based biosensor methods involving the D29 mycobacteriophage, or other more recently discovered mycobacteriophages that can or may be able to infect MAP (35–37), would not be considered a priority in relation to Johne's disease diagnosis. Veterinary diagnostic laboratories are unlikely to want to invest in expensive biosensor equipment that may require more skilled and knowledgeable operators.

## Author Contributions

The author confirms being the sole contributor of this work and has approved it for publication.

## Conflict of Interest

The author declares that the phagomagnetic (PhMS)-qPCR assay discussed was developed in her laboratory and is patent pending (Grant and Foddai, PCT/EP2020/076632).
