# Supplementary material for: Bacteriophage-Based Methods for Detection of Viable Mycobacterium avium subsp. paratuberculosis and Their Potential for Diagnosis of Johne's Disease
Source: Front Vet Sci. 2021 Mar 11;8:632498. doi: 10.3389/fvets.2021.632498 (PMC7991384; doi:10.3389/fvets.2021.632498)
Supplement: Supplementary file 1 [file Table_1.DOCX]

**Supplementary Table 1.** Comparison of the steps required for the Actiphage® Rapid and PhMS-qPCR one-day assays when testing 50 ml of milk for viable MAP. Information obtained from the two original publications and the Actiphage® Rapid Milk Test Kit instructions..

|  | **Actiphage® Rapid Assay** (Swift and Rees, 2019) | **Phagomagnetic (PhMS)-qPCR**  (Foddai and Grant, 2020) |
| --- | --- | --- |
| **Step 1** | Warm up 50 ml milk samples at room temperature (18–25 °C) for ≥1 h | Warm up 50 ml milk samples in waterbath at 37^o^C for 15 min |
| **Step 2** | Centrifuge milk, wash pellet once in Actiphage® Medium Plus^b^, centrifuge again and resuspend pellet in 200 µl  Actiphage® reagent^b^ | Centrifuge milk and resuspend pellet fraction in 1 ml PBS-T20^a^ |
| **Step 3** | Transfer samples to  microcentrifuge tubes | Transfer samples to  BeadRetriever tube strips |
| **Step 4** | Incubate samples for 3.5 h  in 37^o^C incubator | Perform automated magnetic separation^c^ on 1 ml samples using 15 µl phage-coated beads |
| **Step 5** | Centrifuge samples to sediment lysed MAP cell debris | Transfer 50 µl bead-cell samples to  microcentrifuge tubes |
| **Step 6** | Transfer supernatant to  Rapid (filter) tubes^b^ | Incubate samples for 4 h  in 37^o^C incubator |
| **Step 7** | Centrifuge to obtain DNA in flow-through (~200 µl) | Place tubes in heating block  at 55^o^C for 1 min |
| **Step 8** | Clean up and concentrate DNA  using extraction columns^d^ | Centrifuge samples to sediment  lysed MAP cell debris and clarify  DNA (~50 µl) |
| **Step 9** | Use DNA for IS900 PCR  (or store frozen until required) | Use DNA directly for Taqman  IS900 qPCR (or store frozen until required) |

^a^ PBS-T20, Phosphate buffered saline containing 0.05% Tween 20.

^b^ supplied as part of Actiphage Rapid Assay kit.

^c^ BeadRetriever Environmental programme includes two washes in PBS-T20 and final resuspension of bead-cell complexes in 50 µl 7H9-OADC-2 mM CaCl_2_ broth.
